# The epistemic and aleatory uncertainties of the ETAS-type models: an application to the Central Italy seismicity

**DOI:** 10.1038/s41598-017-11925-3

**Published:** 2017-09-18

**Authors:** A. M. Lombardi

**Affiliations:** Istituto Nazionale di Geofisica e Vulcanologia, Rome, Italy

## Abstract

Stochastic models provide quantitative evaluations about the occurrence of earthquakes. A basic component of this type of models are the uncertainties in defining main features of an intrinsically random process. Even if, at a very basic level, any attempting to distinguish between types of uncertainty is questionable, an usual way to deal with this topic is to separate epistemic uncertainty, due to lack of knowledge, from aleatory variability, due to randomness. In the present study this problem is addressed in the narrow context of short-term modeling of earthquakes and, specifically, of ETAS modeling. By mean of an application of a specific version of the ETAS model to seismicity of Central Italy, recently struck by a sequence with a main event of Mw6.5, the aleatory and epistemic (parametric) uncertainty are separated and quantified. The main result of the paper is that the parametric uncertainty of the ETAS-type model, adopted here, is much lower than the aleatory variability in the process. This result points out two main aspects: an analyst has good chances to set the ETAS-type models, but he may retrospectively describe and forecast the earthquake occurrences with still limited precision and accuracy.

## Introduction

Earthquake engineers and seismologists commonly distinguish between two categories of uncertainty in seismic hazard analysis^1–3^. Aleatory variability is the natural randomness in a process; it is supposed irreducible and inherent natural to the process involved. The epistemic uncertainty is the scientific uncertainty in the model of the process; it is supposedly reducible with better knowledge, since it is not inherent in the real-world process under consideration. Nevertheless, the distinction between aleatory variability and epistemic uncertainty, not commonly used in other fields, can get confusing^3^. In the extreme example in which there is no aleatory variability and the earthquake process is in theory fully knowable, there is only epistemic uncertainty, due to lack of knowledge and limited data. This can be reduced in time, if more data are collected and new models are developed. In the more general case, we do not know what is potentially knowable long in the future and the aleatory variability is determined in the context of the models and of their parameterization. In this way, we include in the aleatory variability what is potentially knowable and, vice versa, the aleatory variability can be reduced as additional parameters are added to the model. Finally, the specific model or range of models, in which the aleatory and epistemic uncertainties are discussed, do not cover the effective epistemic uncertainty. Therefore, this last is underestimated and could increase, not decrease, when additional data have been collected and models developed. By considering what said above, the terms epistemic and aleatory will be put in inverted commas, in the following.

In the small context of Epidemic Type Aftershocks Sequences (ETAS) models^4^, the “aleatory” and “epistemic” uncertainties are well represented in the intensity function. This last is the combination of two main factors: a) the model parameters, including the spatial background distribution, which represents the uncertainty about the specific ETAS-type model and b) the past history, which represents the natural variability of the earthquake occurrence, given the model (see following section for details). The main aim of this paper is to discuss and to quantify the “aleatory” and “epistemic” uncertainties, for a temporal-magnitude-spatial (TMS) ETAS-type model, by a specific application to recent seismicity of Central Italy. In this way, we may quantify accuracy and precision of the ETAS-type models, both in describing the observed seismicity and in forecasting future earthquakes.

## Data and Model

Locations and magnitudes of earthquakes, recorded by the stations of the Italian National Seismic Network (downloadable from cnt.rm.ingv.it), are evaluated in real-time in the surveillance room of the Istituto Nazionale di Geofisica e Vulcanologia (INGV) in Rome and then revised by the analysts of the Italian Seismic Bulletin (Bollettino Sismico Italiano - BSI). By following the BSI strategies of revision, all events with ML ≥ 3.5 are quickly revised, whereas the standard review is done for the smaller events, within an agreed timeframe^5^.

The recent Central-Italy seismicity includes two main sequences: the 2009 L’Aquila sequence, having 5 events above ML5.0 (the largest ML5.9 event occurred on April 06 2009) and the still ongoing 2016–2017 Central Italy sequence, for now having 9 events above ML5.0 and 2 events above ML6.0 (the ML6.0 Amatrice event, at August 24 2016, initiating the sequence, and the Mw6.5 Norcia event, at October 30 2016). At present (2017 August), all events above ML3.5 have been revised, whereas the smaller ones, occurred after August 24 2016, are currently under revision.

**Figure 1:**
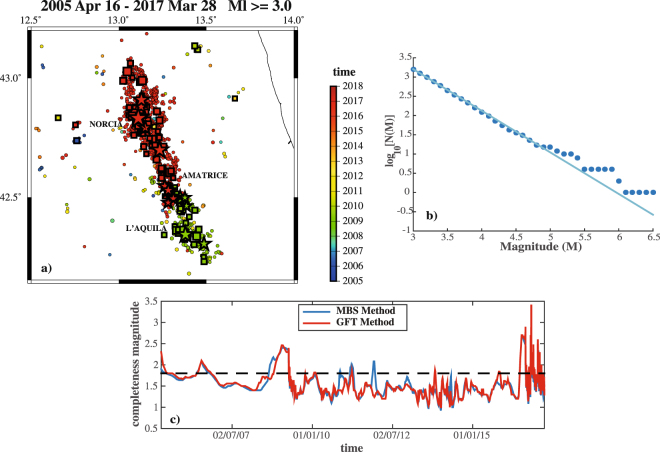
Seismicity occurred in Central Italy from April 16 2005 to March 28 2017. (**a**) Map of events with magnitude above 3.0 and depth between 0 and 40 km (1599 events). Circles, square and stars mark the events with magnitude above 3.0, 4.0 and 5.0, respectively. Size of symbols scales with magnitude, whereas the color changes with the occurrence time. (**b**) Gutenber-Richter Law for the overall catalog. The b-value is equal to 1.1. (**c**) Time-dependent estimation of the completeness magnitude *M*_*c*_ (see text for details). The dotted black line marks the *M*_*c*_ value (1.8) estimated for the overall catalog.

The dataset ${\mathscr{D}}$, analyzed here, collects the events occurred from April 16 2005 up to March 28 2017, in the area [12.50–14.00E, 42.00–43.20N], with magnitude above 3.0 and depth between 0 and 40 km (1,599 events; see Fig. 1a). The starting date marks the start-up of a new seismic network, causing a significant improvement of the earthquakes detection^6,7^. The magnitudes distribution is in agreement with a Gutenberg-Richer Law, having a b-value *b* equal to 1.1 (see Fig. 1b). A time-dependent evaluation of the completeness magnitude (*Mc*), by mean of the Mc and B-value Stability (MBS)^8,9^ and the Goodness of Fit Test (GFT)^10^ methods, reveals a sudden increase of *Mc*, up to ML3.5, soon after the Mw6.5 event (see Fig. 1c). Otherwise, ${\mathscr{D}}$ can be considered complete.

I use the TMS version of the ETAS model (${\mathcal M}$) implemented in SEDAv1.0 (Statistical Earthquake Data Analysis), a statistical software freely provided via the Zenodo open access platform^11^ (https://zenodo.org/record/55277). This has the intensity function

1 $$\begin{array}{rcl}{\lambda }_{TMS}^{ {\mathcal M} }(t,m,x,y/{ {\mathcal H} }_{t}) & = & {\lambda }_{TMS}(t,x,y/{ {\mathcal H} }_{t})\,f(m)\\  & = & [\mu \cdot u(x,y)+\sum _{{T}_{i} < t}\,{e}^{\alpha ({M}_{i}-{M}_{c})}g(t-{T}_{i})h({r}_{i};{M}_{i})]\,f(m)\\  & = & [\mu \cdot u(x,y)+\sum _{{T}_{i} < t}\,{e}^{\alpha ({M}_{i}-{M}_{c})}\tfrac{k}{{(t-{T}_{i}+c)}^{p}}\tfrac{{c}_{d,q,\gamma }({M}_{i})}{{[{r}_{i}^{2}+{d}^{2}{e}^{2\gamma ({M}_{i}-{M}_{c})}]}^{q}}]\\  &  & \times \tfrac{\beta {e}^{-\beta (m-{M}_{c})}}{1-{e}^{-\beta ({M}_{max}-{M}_{c})}}\end{array}$$

where

- ${ {\mathcal H} }_{t}=\{({T}_{i},{M}_{i},{X}_{i},{Y}_{i}),{T}_{i} < t\}$ is the observed data (history) up the time *t*;
- *T*_*i*_, *M*_*i*_ and (*X*_*i*_, *Y*_*i*_) are the time, the magnitude and the epicentral coordinates of the *i*-th event, respectively;
- (*x*, *y*) belongs to a region ${\mathcal R}$;
- $\{u(x,y),(x,y)\in  {\mathcal R} \}$ is the spatial probability density function (PDF) of the background events;
- ${\mathscr{P}}=\{\mu ,k,p,c,\alpha ,d,q,\gamma \}$ are 8 parameters to be estimated, where *μ* is the Poisson background seismic rate, *k* is the productivity coefficient, *p* and *c* are the parameters of the generalized Omori Law, *α* is the coefficient of the exponential magnitude productivity law (called Utsu Law), *d*, *q* and *γ* are the parameters of the spatial distribution of triggered events.
- *r*_*i*_ is the distance (in kms) between the location (*x*, *y*) and the epicenter of the *i*-th event (*X*_*i*_, *Y*_*i*_);
- ${c}_{d,q,\gamma }({M}_{i})=\frac{q-1}{\pi }\,{[{d}^{2}{e}^{2\gamma ({M}_{i}-{M}_{c})}]}^{q-1}$ is a normalization constant, depending on parameters (*d*, *q*, *γ*), so that ${\int }_{0}^{\infty }\,{\int }_{0}^{\infty }\,\frac{{c}_{d,q,\gamma }({M}_{i})}{{[{r}_{i}^{2}+{d}^{2}{e}^{2\gamma ({M}_{i}-{M}_{c})}]}^{q}}dxdy=1,\,\forall i$.

The background spatial PDF *u*(*x*, *y*) is assumed uniform in each of the *N*_*c*_ cells *C*_*j*_ (of area *A*_*j*_) of a regular grid (in degrees), recovering the region ${\mathcal R}$ of interest, so that

2 $$u(x,y)=\sum _{j=1}^{{N}_{c}}\,\frac{{u}_{j}}{{A}_{j}}{1}_{\{(x,y)\in {C}_{j}\}}\quad {\rm{with}}\,\sum _{j=1}^{{N}_{c}}\,{u}_{j}=1$$

where *u*_*j*_ is the probability to observe a background event inside the cell *C*_*j*_, per day.

**Figure 2:**
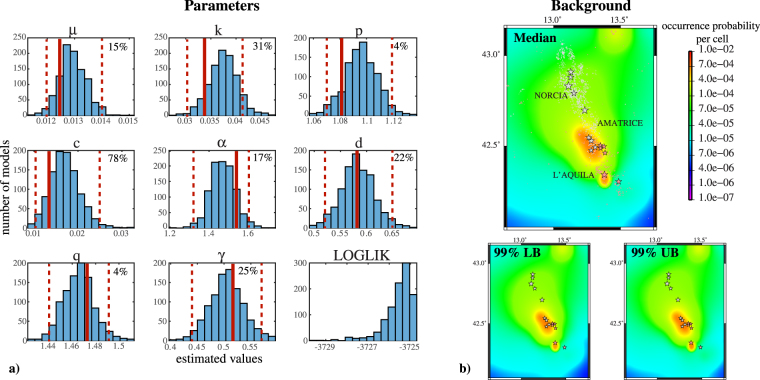
Estimation of the ETAS model (see eq. 1) and of its “epistemic” uncertainty. (**a**) Histograms of values of 8 parameters ${\mathscr{P}}=\{\mu ,\,k,\,p,\,c,\,\alpha ,\,d,\,q,\,\gamma \}$, obtained by running 1000 times a Simulated Annealing algorithm for the log-likelihood optimization (see text for details). The solid red lines mark the parameters values for the model ${ {\mathcal M} }^{best}$, having the largest likelihood. The dotted red lines mark the 95% confidence bounds. The percentages indicate the ratio between the 95% confidence interval size and the median, multiplied by 100, for each parameter. The last panel shows the log likelihood distribution for the 1000 model configurations ${ {\mathcal M} }_{i}$. (**b**) Maps of median and of 99% confidence bounds for the background spatial density function *u*(*x*, *y*), obtained from the 1000 model configurations ${ {\mathcal M} }_{i}$. Pink and white symbols mark the events before and after August 24 2016, date starting the 2016–2017 still ongoing sequence. The stars mark the events with magnitude above 5.0.

I estimate the ETAS model by using the data of ${\mathscr{D}}$ occurred from April 16 2005 to August 24 2016, since these are complete and totally revised by BSI. For the present study, I use a background grid covering the region ${\mathcal R}$ = [12.65–13.75E, 42.05–43.15N] and square cells *C*_*j*_ with a side of 0.01°. By applying the SEDAv1.0 simulated annealing algorithm (1000 runs), based on the maximum likelihood criterion^11,12^, I found the parameters shown in Fig. 2. These distributions represent the “epistemic” uncertainty, in the context of this ETAS-type model, but they are not independent, since some parameters are highly correlated, causing the multimodality of log-likelihood function^13^. The different runs generate similar spatial background distribution (Fig. 2b), whereas some parameters are much more uncertain. Indeed, the proportion of 95% confidence interval size for each parameter, respect to median value (multiplied by 100 to obtain percentages), goes from 4% to 78% (Fig. 2a). In the following, I fix *M*_*max*_ = 7.5, which is a precautionary limit of what expected in Central Italy, by historical and geological data^14,15^. The impact of this choice will be analyzed in the future.

**Figure 3:**
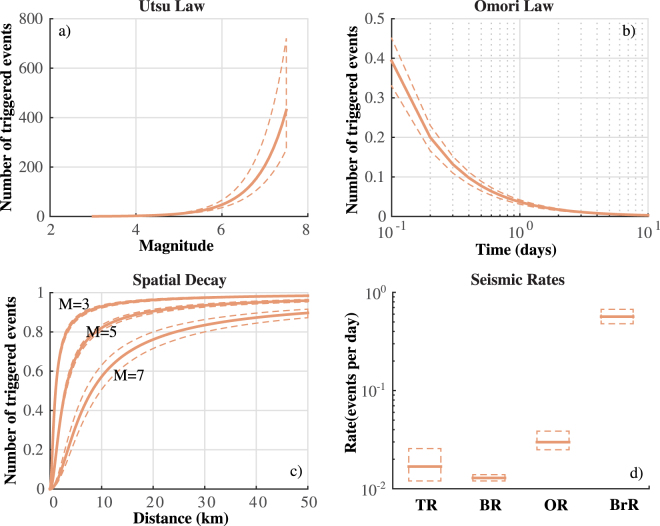
Impact of ETAS model “epistemic” uncertainty on quantitative measures of seismic rate. The solid and dashed lines mark the median and the 99% confidence bounds, respectively. (**a**) Number of directly triggered events versus the magnitude of triggering event. (**b**) Temporal decay of the Omori law *g*(*t*) (see eq. 1). (**c**) Spatial decay of the triggering rate *h*(*r*; *M*) for magnitude 3.0, 5.0 and 7.0 (see eq. 1). (**d**) Variability of rates to equilibrium for the triggered (TR), background (BR) and overall (OR) seismicity and of branching ratio (BrR), for the 1000 configurations of the ETAS model ${ {\mathcal M} }_{i}$.

Figure 3 shows the effects of the model uncertainty on some quantitative measures of earthquake rate. The number of events *N*(*M*) directly triggered by an earthquake of magnitude M (Utsu Law; Fig. 3a), computed by equation

3 $$N(M)={e}^{\alpha (M-{M}_{c})}\,{\int }_{0}^{\infty }\,g(t)dt{\int }_{0}^{\infty }\,{\int }_{0}^{\infty }\,h(r;M)dxdy,$$

does not significantly change with the model parameters, except for *M* close to *M*_*max*_. Negligible changes are found for the functions *g*(*t*) (Omori Law) and *h*(*r*; *M*) (see eq. 1), describing the decay of triggering rate in time (Fig. 3b) and space (Fig. 3c).

Finally, given the branching ratio (expected number of triggered events per mainshock)^16^

4 $$\rho ={\int }_{0}^{\infty }\,{\int }_{{M}_{c}}^{{M}_{max}}\,{e}^{\alpha (M-{M}_{c})}g(t)\,f(m)dtdm=\frac{k\cdot {c}^{p-1}}{p-1}\frac{1-{e}^{-(\beta -\alpha )({M}_{max}-{M}_{c})}}{1-{e}^{-\beta ({M}_{max}-{M}_{c})}},$$

which has a 95% confidence interval size equal to about 35% of its median value (0.57), we can compute the background (*BR*), triggering (*TR*) and overall (*OR*) rates to equilibrium, for each model, by equations

5 $$BR=\mu \quad TR=\frac{\mu \rho }{1-\rho }\quad OR=BR+TR=\frac{\mu }{1-\rho }.$$

whereas *BR* varies in a range equal to about 15% (95% confidence level; see Figs 2 and 3d) of its median value, the respective percentages for *TR* and *OR* are close to 80% and 50% (Fig. 3d).

## The “epistemic” and “aleatory” uncertainty

As above said, the intensity function of the ETAS-type models (eq. 1) well represents and separate the “aleatory” and “epistemic” uncertainties. The history ${ {\mathcal H} }_{t}$ refers to “aleatory” natural randomness of the process, whereas the parameters ${\mathscr{P}}$, together with the spatial background distribution *u*(*x*, *y*), represent the “epistemic” reducible uncertainties.

To quantitatively measures both “aleatory” and “epistemic” uncertainties, two types of analysis will presented in the following. In the first, the history is kept fixed to the observations ${ {\mathcal H} }_{t}^{obs}$, whereas the uncertainty of parameters is token into account. The second analysis consists in fixing the model to the best one ${ {\mathcal M} }^{best}$ (having the maximum likelihood; see Fig. 2, red lines) and in studying the “aleatory” variability by mean of simulations. For each of two analyses, both the expected temporal and the spatial distributions of earthquakes are investigated.

### “Epistemic” uncertainty: effects of parameters variation

In this analysis the history is kept fixed to to ${ {\mathcal H} }_{t}^{obs}$ and the model variability is token into account. For each of 1000 model configurations ${ {\mathcal M} }_{i}$ and for each day *D*_*j*_, the number of events computed by the model $N{e}_{th}^{i,j}$ is compared with daily observations ${N}_{obs}^{j}$, registered in the database ${\mathscr{D}}$. The theoretical daily numbers of events $N{e}_{th}^{i,j}$ are computed by integrating the intensity function (see eq. 1) on the overall region ${\mathcal R}$, the entire magnitude range [*M*_*c*_, *M*_*max*_] and the interval time *D*_*j*_

6 $$N{e}_{th}^{i,j}={\int }_{{D}_{j}}\,{\int }_{ {\mathcal R} }\,{\int }_{{M}_{c}}^{{M}_{max}}\,{\lambda }_{TMS}^{{ {\mathcal M} }_{i}}(t,m,x,y/{ {\mathcal H} }_{t}^{obs})dt,dx,dy,dm$$

where ${\lambda }_{TMS}^{{ {\mathcal M} }_{i}}(t,m,x,y/{ {\mathcal H} }_{t}^{obs})$ is obtained by eq. 1, model ${ {\mathcal M} }_{i}$ and history ${ {\mathcal H} }_{t}^{obs}$.

**Figure 4:**
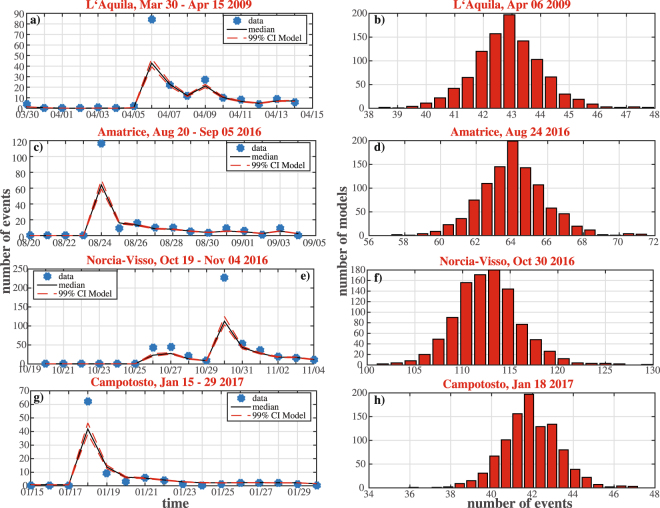
“Epistemic” uncertainty for the ETAS-type models. (**a**) Comparison between the theoretical and the observed daily number of events for a time window of 15 days, including the main event of the 2009 L’Aquila sequence (ML5.9, April 06 2009). (**b**) Histogram of the theoretical numbers of events, defined by the 1000 model configurations ${ {\mathcal M} }_{i}$, at April 06 2009. (**c**) The same of a) but for the ML6.0 Amatrice event (August 24 2016). (**d**) The same of (**b**) but for August 24 2016. (**e**) The same of (**a**) but for the Mw6.5 Norcia event (October 30 2016). (**f**) The same of (**b**) but for October 30 2016. (**g**) The same of (**a**) but for the ML5.4 Campotosto event (January 18 2017). (**h**) The same of (**b**) but for January 18 2017.

Figure 4(a,c,e,g) shows some time windows of 15 days, including all events above ML5.0. Firstly, the model uncertainty is negligible, since the 99% confidence bounds of theoretical rates $N{e}_{th}^{i,j},\,i=1,\,\ldots ,\,1000\}$ are very close to their median, for each day *D*_*j*_. Secondly, the model generally well describe the time behavior of seismicity, but it is not able to fit the number of events observed at occurrence days of main events. You have to remember that this analysis does not include the natural randomness of the process, so that the observed number of events ${N}_{obs}^{j}$ is a percentile of a random distribution, predicted by ${ {\mathcal M} }_{i}$, having as mean $N{e}_{th}^{i,j}$. Anyway, the systematic underestimation of observed number of events, soon after the occurrence of largest events, suggests an intrinsic inefficiency of the model, probably only partially justified by the preliminarity of data.

Figure 4(b,d,f,h) show the histograms of $N{e}_{th}^{i,j}$ for days *D*_*j*_ in which 4 of largest events occurred (ML5.9 Apr 06 2009 L’Aquila; ML6.0 Aug 24 2016 Amatrice; Mw 6.5 Oct 30 2016 Norcia; ML 5.4 Jan 18 2017 Campotosto). These histograms represent the “epistemic” uncertainty on the theoretical number of events. All these distributions have a variance well lower the median, showing that impact of model variability is negligible.

**Figure 5:**
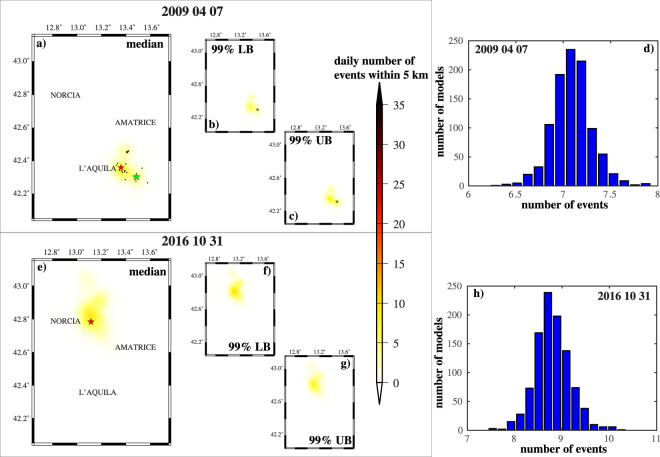
“Epistemic” uncertainty on the spatial distribution of events, predicted by the ETAS-type models. (**a**) Median spatial distribution of events at April 07 2009, computed by the 1000 ETAS model configurations ${ {\mathcal M} }_{i}$. The values represents the theoretical number of events within 5 km from the cell center. The black points and green stars mark the events above 3.0 (22 events) and 5.0, respectively, occurred at April 07 2009. The red star marks the main event of the 2009 sequence (ML 5.9; April 06 2009). (**b**) The same of (**a**) but for 99% lower confidence bound. (**c**) The same of (**a**) but for 99% upper confidence bound. (**d**) Distribution of the theoretical number of events at April 07 2009, in the cell including the main event of the 2009 L’Aquila sequence (ML5.9, April 06 2009). (**e**) The same of (**a**) but for October 31 2016. The number of events with magnitude above 3.0 (black points) is 54. The red star marks the main event of the 2016–2017 sequence (Mw 6.5; October 30 2016). (**f**) The same of (**b**) but for October 31 2016. (**g**) The same of (**c**) but for October 31 2016. (**e**,**h**) The same of (**d**) but for October 31 2016 and for the cells including the main event of the 2016–2017 Central Italy sequence (Mw6.5, October 30 2016).

Figure 5 shows a quantitative evaluation of the spatial variability of theoretical seismic rate, at April 07 2009 and October 31 2016, the days after the occurrence of strongest events of 2009 and 2016–2017 sequences. Specifically, for each model ${ {\mathcal M} }_{i}$, l compute the theoretical daily number of events within 5 km from the center of each cell, in order to minimize the impact of possible locations errors of not revised events. The maps of the median and of the 99% confidence bounds of these rates, for each cell, show that the spatial variability is negligible, including the areas near the locations of main events (Fig. 5d,h).

### “Aleatory” uncertainty: the natural randomness

In this analysis the model is fixed to ${ {\mathcal M} }^{best}$, having the maximum log-likelihood (see Fig. 2, red lines), and the history is changed by simulations. Specifically, for each day *D*_*j*_, the system consider 10000 different histories ${ {\mathcal H} }_{t}^{k,j};\,k=1,\,\ldots ,\,10000$, consisting of observations occurred before *D*_*j*_ (so that all histories are equal before *D*_*j*_) and of simulations inside *D*_*j*_. These simulations corresponds to what the model ${ {\mathcal M} }^{best}$ forecasts, given the observed history ${ {\mathcal H} }_{t}^{obs}$, before *D*_*j*_. The theoretical daily number of events $N{a}_{th}^{k,j}$ are computed by integrating the intensity function on the overall region, the entire magnitude range and the interval time *D*_*j*_, for each history ${ {\mathcal H} }_{t}^{k,j}$

7 $$N{a}_{th}^{k,j}={\int }_{{T}_{j}}\,{\int }_{ {\mathcal R} }\,{\int }_{{M}_{c}}^{{M}_{max}}\,{\lambda }_{TMS}^{{ {\mathcal M} }^{best}}(t,m,x,y/{ {\mathcal H} }_{t}^{k,j})\,dt,dx,dy,dm.$$

**Figure 6:**
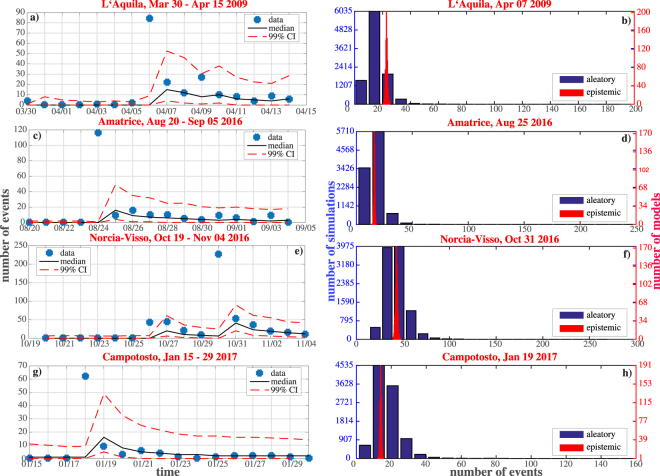
The same of Fig. 4, but for the “aleatory” uncertainties. The blue histograms show the distribution of the theoretical numbers of events at days after the occurrence of some of strongest events of both 2009 and 2016–2017 sequences, obtained from the 10000 earthquake simulations (see text for details). The red histograms show the “epistemic” uncertainty, defined by the 1000 model configurations ${ {\mathcal M} }_{i}$, similarly to Fig. 4.

Figure 6 corresponds to Fig. 4, for this second analysis. The first result is that the “aleatory” uncertainty is much larger than the “epistemic” one. Specifically, the distribution of occurrences is asymmetric, with a strongly positive skewness. This result is due to the not negligible probability to have a medium-large event, causing an increase of seismic rate. Secondly, the observed number of events fully falls within the range of model forecasts, but for days in which the main events occur, since these are not still included in the histories. This means that what actually happened is generally covered by the model ${ {\mathcal M} }^{best}$.

**Figure 7:**
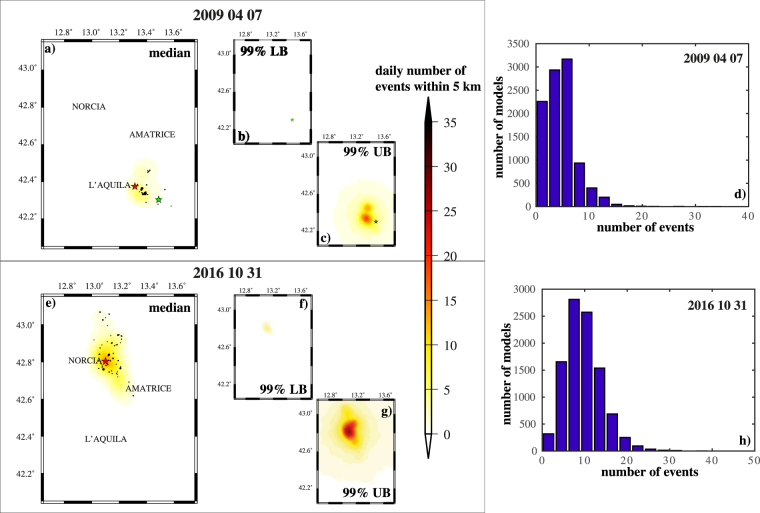
The same of Fig. 5, but for the “aleatory” uncertainties. The maps and histograms refers to theoretical distribution of events at days after the occurrence of mainshocks of 2009 (L’Aquila) and 2016–2017 (Central Italy) sequences.

The check of the spatial variability of the seismic rates (Fig. 7) show a much larger natural randomness respect to the “epistemic” uncertainty (Fig. 5), especially around the main event location. This is much more close to a binomial distribution than to a poisson one, since it has a variation much lower than the median and a high skewness.

## Discussion and Conclusions

The main aim of this paper is to quantitatively measure uncertainty of an ETAS-type model. The role of uncertainties and the manner to address them appropriately has been discussed by different specialists for a long time (see ref.^17^). The nature of uncertainties of a model and how one deals with them depends on the context and the application. Taking in mind the questionable meaning of terms “epistemic” and “aleatory” (as discussed in the Introduction section), these measures may be useful to improve the accuracy of a model and to provide an objective judgment of its both retrospective and forecast performances. Specifically, this paper presents results deriving by the application of an ETAS-type model on Central Italy seismicity.

The main result of this paper is that the “aleatory” variability is much larger than the “epistemic” uncertainty, at least in the context of the specific version of the ETAS-type model used here. This result has two important consequences. The first is that the Simulated Annealing algorithm, used to set the model, has a good search ability, which allows to return the optimal solution of the log-likelihood maximization. Different sets of parameters may lead to very close log-likelihood, due to strong correlation among them. This result partially invalidates the physical meaning given to individual parameters, but allows us to quantify their resolution. Whereas the speeds of the temporal and spatial decay for the triggered events (*p* and *q* parameters) are well constrained, *c* parameter is much more uncertain (Fig. 2). The moderate variability of background rate (Fig. 2a) and of background spatial distribution (Fig. 2b) points out a good discrimination between independent and triggered events. This is confirmed by low variability of background equilibrium rate *BR* and of branching ratio *BrR* (Fig. 3d). More generally, the parameters variability has a small impact on the main features of the model (Fig. 3).

The analysis of the “epistemic” uncertainty, discussed in the present paper, comprises only the parameters uncertainty of the ETAS-type model proposed here. None consideration is made about different parametrization of the ETAS modeling or about further short-term stochastic models. The parametric uncertainty analysis (Figs 4 and 5) shows a good ability of the model to retrospective forecast the occurrences, considering that the variance of what expected is not taken into account. A quantitative test of retrospective agreement with observation is beyond the scope of this paper and will be made in the future. In the context of present study, the sensitivity analysis on model parameters is not conducted, due to correlations among them^13^. Indeed, the distributions shown in Fig. 2 are not independent and cannot be used individually, for the identification of more critical parameters. The right way forward is to include these correlations by using model configurations ${ {\mathcal M} }_{i}$.

The large aleatory uncertainty (Figs 6 and 7), so as the scarce ability to retrospectively fit observations soon after the strongest events (Fig. 4), is associated with the still poor modeling of main short term features of seismicity. The ETAS-type models do not take into account specific characteristics of the ongoing seismicity, as the possible activation of close major faults, during a sequence. The background rate, constant in time and variable in space, is inadequate to represent the complexity of long-term physical processes, leading the starting of significant sequences. For these reasons, the ETAS-type models are able to provide plausible, even if highly uncertain, forecasts of aftershocks, but they do not usefully predict the occurrence of large shocks, both before and during a sequence. All these results show that a great effort needs to be done to improve this type of models.

The present paper does not discuss the impact of data uncertainty and of highly temporary incompleteness, soon after the Mw6.5 event occurrence (October 30 2016). This topic will be treated in future work, when the catalog revision by BSI will be finished. This is of prominent importance for two reasons. Firstly, data uncertainty is a particular type of epistemic uncertainty that might play a role in judging the performance of ETAS-type models^18^. Secondly, the assessment of data uncertainty/incompleteness, and of their impact on the ETAS-type models, can help to provide new, more efficient, real time forecasting procedures, comprised in the Operational Earthquake Forecasting^19^.

The impact of incomplete data set on the ETAS model estimation has been studied by Omi, Ogata and colleagues^20–22^, which proposed a Bayesian estimation method to apply on incomplete early aftershock data, soon after the occurrence of larges events. Anyway, in the present study, the temporary incompleteness is a problem of lesser importance, since it is limited to the first day after the main event occurrence (Mw6.5, October 20, 2016; see Fig. 1) and the model is estimated on data occurred before the 2016–2017 Central Italy sequence (up to August 24 2016).

The shape of aleatory uncertainty (Figs 6 and 7) is more close to a binomial distribution than a poisson one. This result does not confirm that a binomial distribution always well approximates forecasts made by an ETAS-type models. This topic will be investigated in future work. Nevertheless, this result is in agreement with the well-known result of unsuitability of Poisson hypothesis for this type of forecasts^23,24^.

Aleatory uncertainty is naturally associated with the uncertain future of the system (i.e., the occurrence of the events). Anyway, as above said, it is not independent by not-aleatory uncertainties. Reducing aleatory variability might be not inexpensive and causes an increase of epistemic uncertainty. This last includes a “blind” ignorance, more difficult to deal with because of their unknown nature^25,26^. Even if the classification of uncertainty as aleatory or epistemic is ambiguous, studies like this are likely to reveal important information about the reliability of existing models and to provide guidance on how they can be improved.
